# Carbon Dioxide Selectivity over Ethane in Promising Bis Tri (Fluoromethylsulfonyl) Imide-Based Ionic Liquids

**DOI:** 10.3390/molecules30050984

**Published:** 2025-02-20

**Authors:** Eric Quaye, Amr Henni, Ezeddin Shirif

**Affiliations:** 1 Industrial Systems Engineering, University of Regina, Regina, SK S4S 0A2, Canada; enq014@uregina.ca; 2 Energy Systems Engineering, University of Regina, Regina, SK S4S 0A2, Canada; ezeddin.shirif@uregina.ca

**Keywords:** ethane, intelligent gravimetric analyzer (IGA), ionic liquids, CO_2_ capture

## Abstract

This research addresses the critical challenge of CO_2_ capture by exploring innovative ways to avoid ethane (C_2_H_6_) co-absorption in natural gas sweetening operations. The solubility of Ethane (C_2_H_6_) was measured in three ionic liquids (ILs) with similar anions, 1-decyl-3-methyl imidazolium bis (trifluoro methylsulfonyl imide) [IL-1], 1-hexadecyl-3-methylimidazolium bis (trifluoro methylsulfonyl imide) [IL-2], and triethytetra-decyl ammonium bis (trifluoromethylsulfonyl imide) [IL-3]. The solubility experiments were investigated at 303.15 K and 343.15 K with pressures reaching 1.2 MPa. Among the ILs, [IL-2] exhibited the highest ethane absorption capacity due to its extended alkyl chain. The Peng-Robinson equation of state (PR-EoS) and three (3) distinct mixing rules provided robust correlations for the solubility data. Results demonstrate the inferior performance of [IL-1], [IL-2], and [IL-3] compared to Selexol/Genosorb 1753. The selectivity of Ethane (C_2_H_6_) over CO_2_ was determined, with the overall selectivity ranking as follows: [IL-1] > [IL-3] > [IL-2]. A comparison of these selectivity values with published IL data indicated that these three ILs are most effective when used in applications targeting CO_2_ capture in the absence of Ethane (C_2_H_6_), such as in the case of flue gas. They will most probably be used with an amine blend. Additionally, the Enthalpy and entropy of absorption provided valuable insights, demonstrating Ethane’s weaker interactions and lower solubility than CO_2_. These findings emphasize the critical role of IL structure in determining ethane solubility and highlight the potential of customized ILs for optimizing gas-separation processes.

## 1. Introduction

Since the Industrial Revolution, the steady rise in greenhouse gas (GHG) emissions, particularly carbon dioxide (CO_2_), has posed a significant challenge to global climate stability. Industrial and energy sectors have been primary contributors, as emissions increased with economic growth and energy demand. For Peng et al. [1], this continuous escalation has created a global dilemma requiring urgent and innovative approaches to reduce such emissions and move towards carbon neutrality. While efforts to mitigate carbon dioxide emissions have gained considerable attention, such as Carbon Capture and Utilization and Sequestration (CCUS), other critical components of natural gas, such as methane (CH_4_) and Ethane (C_2_H_6_), also warrant focused investigation. Ethane, a key byproduct of natural gas processing and a precursor to ethylene production, represents both a challenge in separation technologies and an opportunity for enhancing industrial sustainability. Addressing these interconnected issues requires a deeper understanding of solvent performance under varying conditions, offering potential pathways for more efficient gas separation and environmental impact mitigation [1].

Ionic liquids (ILs) have garnered considerable interest for their potential across diverse energy applications, such as membranes, proton conductors, and solar cells. Their unique characteristics, including high CO_2_-absorption capacity, low volatility, and biodegradability, position them as viable alternatives, in some cases, to traditional amine-solvent processes, which are often corrosive and volatile [2]. Tailored from organic cations and anions, ILs have melting points below 100 °C, making them adaptable to various applications. Although their usage was initially limited to organic chemistry and electrochemistry, Freemantle’s 1998 study marked a pivotal expansion into green chemistry [3]. This development paved the way for ILs’ broader adoption in biochemistry, catalysis, environmental remediation, enhanced oil recovery, and CO_2_-capture technologies. Their non-flammability and tunable properties not only enhance CO_2_ solubility but also reduce reliance on volatile organic compounds (VOCs), contributing to environmental sustainability. Consequently, research has increasingly highlighted ILs as eco-friendly solvents for advanced carbon-capture processes [4].

Ionic liquids (ILs) are classified into two main categories: Task-Specific Ionic Liquids (TSILs) and Room Temperature Ionic Liquids (RTILs), each offering unique advantages for CO_2_ capture. RTILs function predominantly as physical absorbents, obeying Henry’s Gas Law and providing tunable solubility for CO_2_ without undergoing chemical reactions. In contrast, TSILs exhibit a dual capability, combining chemical and physical solubility, significantly enhancing their efficiency in CO_2_ capture applications. Vadillo et al. [5] highlight the superior performance of TSILs, which arise from tailored functional groups that chemically interact with CO_2_ molecules while maintaining physical absorption properties. This dual functionality positions TSILs as promising candidates for advanced carbon-capture technologies, emphasizing the potential of ILs to bridge the gap between efficiency and environmental sustainability [5]. We performed a molecular simulation study using COSMO-RS [6] that predicted Henry’s Law constants for IL-1 and IL-2 at 25 °C, resulting in 3 MPa and 2.8 MPa values, respectively. In a recent work [7], we obtained the experimental values for Henry’s Laws for these two ILs and found them equal to 3 MPa at 30 °C and 3.66 at 50 °C, respectively. Both studies suggested that these ionic liquids are excellent solvents for CO_2_ capture.

The goal of this study is to evaluate the absorption capabilities of the three (3) ionic liquids (ILs) recognized as promising carbon dioxide physical solvents in absorbing Ethane (C_2_H_6_) across a temperature range of 303.15 K to 343.15 K and pressures up to 1.4 MPa, conditions relevant to industrial applications. The investigation employs an Intelligent Gravimetric Microbalance (IGA-003) to precisely evaluate ethane solubility and assess the suitability of ILs for CO_2_ capture without ethane co-absorption.

## 2. Results

### 2.1. Ethane (C_2_H_6_) Absorption Validation Test

**Figure 1:**
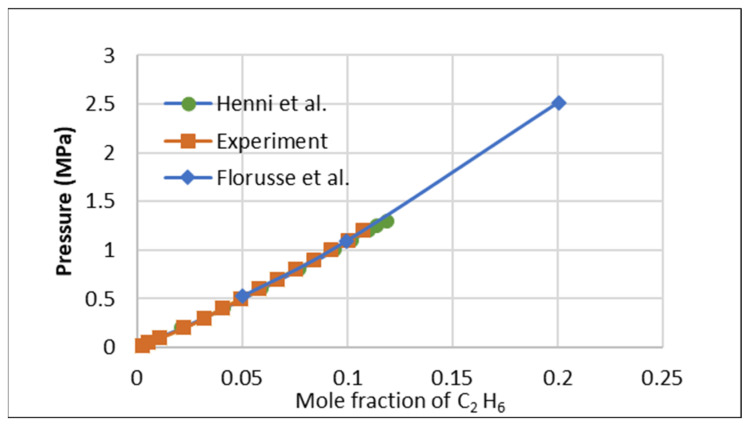
Ethane validation test with Florusse et al. [8] and Henni et al. [9] at 323.15 K.

To evaluate the repeatability of the IGA-003 against other isotherm experiments and its adherence to experimental SOPs, a study was conducted to assess the solubility of C_2_H_6_ in a widely used ionic liquid, [HMIM] [Tf2N]. The collected data were compared with previously published results by Florusse et al. [8] and Henni et al. [9], as presented in Figure 1. This comparison validated the current measurements’ precision and the experimental setup’s reliability.

### 2.2. C_2_H_6_ Solubility in Selected ILs

**Figure 2:**
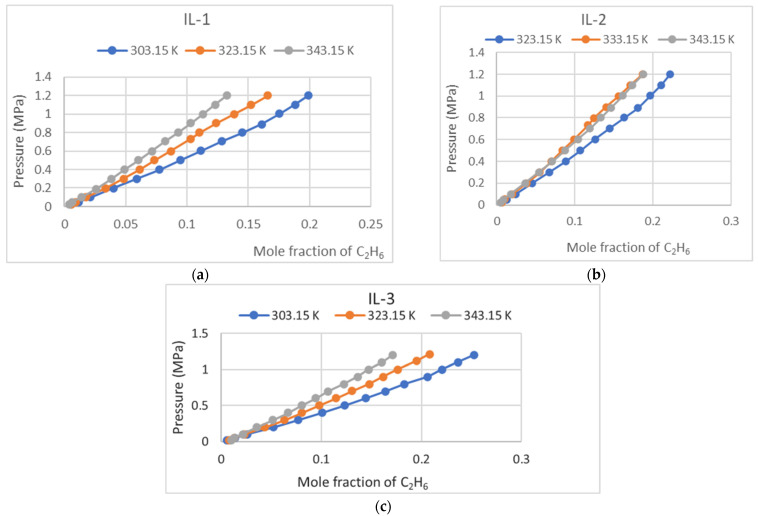
(**a**) C_2_H_6_ absorption in IL-1; (**b**) C_2_H_6_ absorption in IL-2; (**c**) C_2_H_6_ solubility in IL-3.

Ethane (C_2_H_6_) absorption in IL-1, IL-2, and IL-3 was measured under various temperature and pressure conditions, up to a maximum of 1.4 MPa. The collected data were analyzed to observe trends in solubility behavior and are presented graphically in Figure 2 and numerically in Table 1 below. This research provides an understanding of the potential of these ionic liquids for ethane capture applications.

### 2.3. Interaction Parameters Evaluation from Simulation

The data collected in the experiments were regressed using three (3) mixing rules: (a) PR+vdW1, (b) PR+vdW2, and (c) PR+WS+NRTL. Table 2 summarizes the binary interaction parameters calculated for each distinct mixing rule alongside their respective average absolute deviations (AAD%). For ethane (C_2_H_6_) absorption in these ionic liquids (ILs), the following AAD% values were obtained:

- IL-1: vdW1 (3.87%), vdW2 (1.93%), and WS-NRTL (1.96%).
- IL-2: vdW1 (3.63%), vdW2 (1.62%), and WS-NRTL (1.58%).
- IL-3: vdW1 (5.09%), vdW2 (1.58%), and WS-NRTL (1.53%).

The Wong-Sandler (WS-NRTL) mixing rule consistently produced the lowest AAD% for all ILs, underscoring its effectiveness and reliability as the superior choice among the evaluated mixing rules. This suggests that the WS-NRTL approach provides a more accurate representation of ethane absorption in these IL systems.

### 2.4. Henry’s Law Constants, Entropies and Enthalpies of Solvation

Henry’s Law constants (H) were evaluated using a plot of fugacity against ethane mole fraction and correlating the data to a second-order trend line. The gradient/slope derived from this polynomial fit served as the basis for calculating the Henry’s Law constant, a critical parameter for quantifying the gas solubility. Additionally, entropy values were estimated by plotting the natural logarithm (Ln) of Henry’s constant (H) against the natural logarithm (Ln) of its corresponding temperature (T). The analysis revealed negative enthalpy values, characteristic of an exothermic solubility process and indicating an increased disorder upon solution. These results, coupled with the negative entropy values shown in Table 3 below, offer a valuable understanding of the thermodynamic behavior of the ionic liquid during ethane (C_2_H_6_) absorption.

Table 3 highlights a relatively higher ethane (C_2_H_6_) absorption by the ionic liquids (ILs) studied compared to Selexol/Genesorb 1753 [10], a widely used commercial physical solvent in gas treatment applications. Selexol/Genesorb 1753 demonstrates a lower capacity for C_2_H_6_ absorption, making it advantageous for applications where minimal ethane capture is desirable. The ILs, namely IL-1, IL-2, and IL-3, exhibit varying degrees of interaction with Ethane, as reflected by their Henry’s law constants across different temperatures. IL-1, with the highest Henry’s law constants, indicates a lower affinity for Ethane, translating to reduced absorption. Conversely, IL-2 displays the strongest affinity, absorbing more Ethane under similar conditions, as supported by its lower Henry’s law constant values. This variation underscores the distinct thermodynamic and interaction behaviors of these ILs, guiding their potential application in selective gas-separation processes.

Kurnia et al. [11] performed a molecular dynamic study on 16 ionic liquids. They found that bis (trifluoromethyl sulfonyl imide)-based ionic liquids at 25 °C had Henry’s Law constants in Ethane varying from 5.8 MPa for [C10C1im] to 17.8 MPa for [C2C1im]. Two of our ILs had lower values ranging from 4.58 to 4.80 MPa. The authors used the charge distribution, σ (sigma), of the specific polarity on the molecular surface and the likeliness of a compound to a solvent (σ-profile) to screen the ionic liquids. They confirmed that Ethane possesses a nonpolar character and exhibits attraction toward nonpolar groups. The presence of a polar aromatic ring, like in imidazolium (IL-1 and IL-2), helped decrease ethane solubility. The study concluded that ILs have low ethane solubility compared to CO_2_. They also confirmed that increasing the number of carbon atoms attached to the imidazolium ring (longer alkyl chain) increases the nonpolarity and, therefore, the solubility of Ethane. Consequently, we expect IL-2 [C16mim] to absorb more Ethane than IL-1 [C10mim). Our measurements confirm this fact at all temperatures. This observation is confirmed by the positive values of overall polarity (N) reported by Sumon et al. [6], with a value of 49.91 for IL-2 and 41.38 for IL-1, meaning that they are both nonpolar, IL-2 more so than IL-1, as confirmed by the lower Henry’s Law constants.

Kurnia et al. [11] also showed that bis (trifluoromethylsulfonyl) imide anion has a high-intensity peak in the nonpolar region at 0.3 e.nm^−2^. This was confirmed by our study [6], where the value of N for [Tf2N] was found to be positive (6.22), displaying, therefore, nonpolar characteristics. In terms of ranking anions, Kurnia et al. [11] found that among anions, [BF4, tetrafluoroborate] demonstrated the lowest solubility of Ethane, followed by [PF6, hexafluorophosphate], [TFA, trifluoroacetate], [EtSO4, ethylsulfate], [Tf2N, bis-(trifluoro-methylsulfonyl)imide], [DMP, dimethylphosphate,], [Ac, acetate] and [OcSO4, octylsulfate], with the highest solubility in [DBP, dibutylphosphate]. The study showed that bis (trifluoromethylsulfonyl) imide [Tf2N] was in the middle of the list in its ability to absorb Ethane. The fluorinated [Tf2N] anion increases the solubility of CO_2_ but decreases that of Ethane, making these ILs promising for CO_2_ selectivity over Ethane. The authors also found that the solubility of Ethane does not depend only on the polarity as it can have a preferential site in which to interact with the IL.

**Figure 3:**
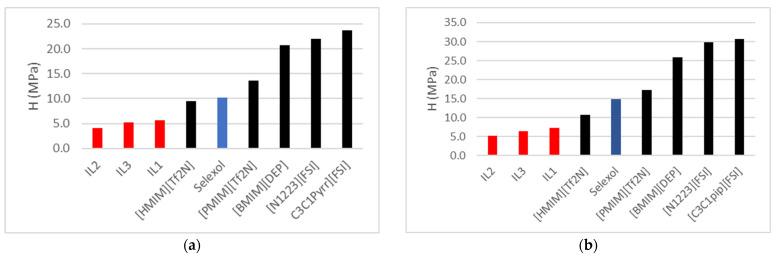
Henry’s Gas law constant (H) comparison for C_2_H_6_ absorption between ILs in this study versus published ILs [9,12] and Selexol/Genesorb 1753 [10] at (**a**) 323.15 K and (**b**) 343.15 K.

Figure 3a,b below compares the ionic liquids (ILs) studied in this research with those documented in the literature for ethane (C_2_H_6_) solubility [12]. This comparison places the current findings in perspective, highlighting the performance of the ILs under study relative to previously published ILs. By juxtaposing the ethane-solubility capacities, the study aims to identify ILs with optimized properties for specific gas-separation applications, showcasing advancements or gaps relative to established benchmarks.

### 2.5. Selectivity-CO_2_/C_2_H_6_

Selectivity parameters are pivotal in designing enhanced separation systems for carbon capture and storage (CCS) applications, improving energy efficiency and minimizing project costs. Moreover, the preferential solubility of CO_2_ over Ethane highlights potential applications for captured CO_2_ in green energy technologies, advancing the move to a more environmentally green future. The selectivity of CO_2_ relative to C_2_H_6_ is estimated using the following relation:

(1) $$S_{{CO}_{2}/C_{2}H_{6}}=\frac{H_{C_{2}H_{6}}}{H_{{CO}_{2}}}$$

The selectivity (CO_2_/C_2_H_6_) for the ionic liquids shown in Table 4 studied in this research is ranked as follows:

IL-1 > IL-3 > IL-2

This ranking indicates that IL1 exhibits the highest selectivity for CO_2_ over C_2_H_6_, making it the most promising candidate for separation processes where minimizing ethane absorption is crucial. Conversely, IL-2 demonstrates the lowest selectivity, suggesting its limited efficacy in such applications compared to IL-1 and IL-3. These insights are critical for optimizing the choice of ILs in gas-separation technologies, particularly for carbon capture and storage (CCS) systems.

**Figure 4:**
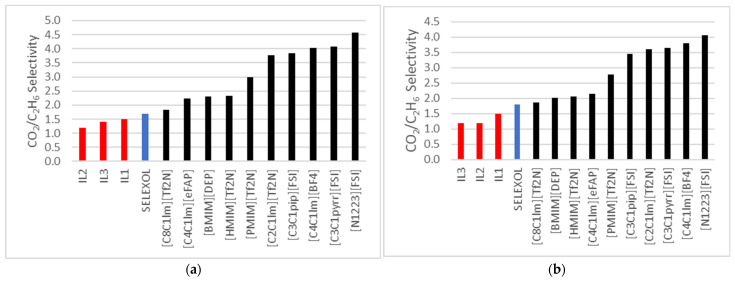
(**a**) Comparison to CO_2_/C_2_H_6_ selectivity data for ILs published by Nath and Henni et al. [12] and Rayer al. [10] at 323 K; (**b**) at 343.15 K.

Figure 4a,b below compares the selectivity (CO_2_/C_2_H_6_) of the ILs in this research with selectivity values reported by Nath and Henni [12] and Rayer et al. [10]. Overall, the ILs examined in this study displayed relatively lower selectivity values across various temperatures than those reported in the literature. This observation highlights potential limitations in the selectivity performance of the ILs tested, providing valuable insights for further optimization in gas-separation applications.

A broader evaluation of the ILs studied in this research, illustrated in the figures below, suggests that their selectivity towards CO_2_ is less pronounced than that of other published ILs. However, these ILs demonstrate excellent potential as solvents in processes where ethane co-absorption is not critical or Ethane is absent in the feed stream. This makes them viable candidates for specialized applications requiring minimal interference from Ethane.

## 3. Discussion

In this research, the absorption behavior of Ethane (C_2_H_6_) in three (3) ionic liquids [1-decyl-3-methylimidazolium bis (trifluoromethylsulfonyl) imide (IL-1), 1-hexadecyl-3-methylimidazolium bis (trifluoromethylsulfonyl) imide (IL-2), and triethyl tetradecyl ammonium bis (trifluoromethylsulfonyl) imide (IL-3)] was studied at a temperature range of 30–70 °C and at industrial-relevant pressure. Henry’s law constants were used to estimate the solubility of Ethane, and the results provided insights into the effect of temperature, molecular structure, and ionic liquid composition on C_2_H_6_ absorption.

### 3.1. Temperature Dependence of Ethane Absorption

The experimental data confirms ethane solubility in the studied ILs decreases with increasing temperature. Henry’s law constants increased across all ILs as the temperature rose from 303.15 K to 343.15 K, indicating reduced absorption. This trend aligns with the exothermic nature of gas absorption in ionic liquids, as suggested by the negative enthalpy values observed in previous studies. Lower temperatures favor ethane absorption due to thermodynamic stability, while at higher temperatures, the ILs tend to reject C_2_H_6_, reducing their capacity as solvents for absorption.

### 3.2. Effect of Ionic Liquid Structure on Ethane Absorption

The three ionic liquids showed varying affinities for Ethane, highlighting the impact of both the cation structure and the alkyl chain length.

### 3.3. Alkyl Chain Length

IL-1 and IL-2 are imidazolium-based ionic liquids with the same anion, but IL-2 contains a longer alkyl chain (hexadecyl) than IL-1 (decyl). The results from the experiment showed that IL-2 showed a relatively higher ethane-absorption capacity than IL-1 across all temperatures. This experimental result can be associated with the high van der Waals interactions delivered by the longer alkyl chain, which enhances the solubility of nonpolar gases such as Ethane. This trend agrees with findings in the literature, where longer alkyl chains generally increase gas absorption due to increased hydrophobicity and greater free volume within the IL structure [12].

### 3.4. Cation Effect

IL-3, which contains a tetradecyl ammonium-based cation, showed intermediate absorption capacity between IL-1 and IL-2. Compared to the imidazolium-based ILs, the ammonium cation introduces subtle changes in the IL’s polarity and free volume, affecting its interaction with ethane molecules. While the ethane solubility in IL-3 is higher than in IL-1, it remains lower than in IL-2, suggesting that the alkyl chain length (hexadecyl in IL-2) has a more pronounced effect on absorption than the type of cation used. These findings are consistent with earlier studies that report a dominant role of the IL’s molecular structure—mainly the alkyl chain length—on gas solubility [12].

### 3.5. Comparison of Ionic Liquids Based on Henry’s Law Constants

The ranking of Henry’s law constants for ethane absorption at all temperatures was found to be as follows: IL-2 < IL-3 < IL-1. This order shows that IL-2 has the highest ethane-absorption capacity while IL-1 has the lowest. IL-1′s lower absorption can be attributed to its shorter alkyl chain and reduced van der Waals interactions. On the other hand, IL-2′s superior absorption performance highlights the positive correlation between longer alkyl chains and ethane solubility.

Increasing the number of carbon atoms attached to the imidazolium ring (longer alkyl chain) increases the nonpolarity and, therefore, the solubility of Ethane.

### 3.6. Comparison to Conventional Solvents

To assess the industrial and broader applicability of the studied ILs, their performance was compared to conventional solvents such as Selexol or Genesorb 1753. Henry’s law constants for Ethane in IL-1, IL-2, and IL-3 were relatively lower than those reported for traditional solvents, indicating higher absorption capacities in the studied ILs. While this demonstrates their potential as alternative solvents, it also highlights a challenge: the co-absorption of Ethane in processes where selective CO_2_ absorption is desired.

### 3.7. Implications for Natural Gas Processing

The ability of ionic liquids to co-absorb Ethane can be a limitation in natural gas processing, where separating CO_2_ from hydrocarbons like Ethane is critical. While IL-2 exhibited the highest ethane absorption, making it less ideal for selective CO_2_ capture, IL-1′s lower affinity for Ethane suggests it could be a better candidate for CO_2_/ethane separation applications relative to the other ILs in this research. Nevertheless, the observed Ethane- solubility trends indicate that further structural optimization of ILs—such as modifying the anion or introducing functional groups—is necessary to enhance their selectivity for CO_2_ over Ethane.

## 4. Methodology and Materials

### 4.1. Materials

Table 5 outlines the specific ionic liquids (ILs) employed in this study, listing their purity, chemical structures, and nomenclature. The three ionic liquids were synthesized by IoLiTec (Ionic Liquids Technologies GmbH, Heilbronn, Germany). These ILs, designed for gas-capture applications, are examined for their ability to capture carbon dioxide (CO_2_), as shown in prior studies, and their effectiveness in capturing Ethane (C_2_H_6_). The structural properties and purity of each IL are crucial in understanding their interaction with CO_2_ and Ethane, highlighting how variations in their molecular makeup influence gas solubility. By comparing the performance of these ILs in capturing both gases, the research aims to provide insights into their potential for use in more complex, real-world applications, such as natural gas- separation processes, where both CO_2_ and Ethane are present.

### 4.2. Density Evaluation of the ILs

**Figure 5:**
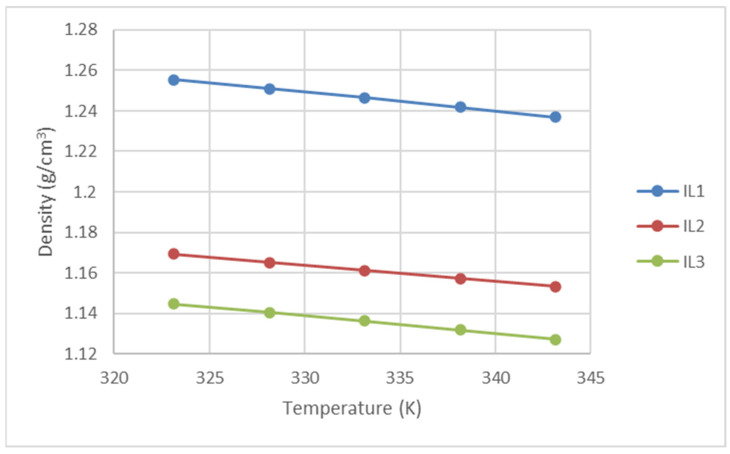
Densities of the ILs used in this work.

To ensure precise density evaluation for the ionic liquids (ILs) in this research, we first measured the specific gravity of n-methyldiethanolamine (MDEA) (purity ≥ 99%) using an Anton Paar density meter. The obtained data values were compared with published values by Karunarathne et al. [13] for MDEA of similar purity, revealing an average absolute deviation (AAD) of just 0.014%. This confirmed the high repeatability and accuracy of our measurements. Subsequently, we measured the densities of three (3) research ILs at atmospheric pressure and temperatures ranging from 303.15K to 343.15K using an Anton Paar Density and Speed of Sound (DSA 5000) meter. The resulting data are presented in Figure 5 and Table 6 below.

### 4.3. Ethane Solubility Analysis

The IGA-003, designed by Hiden Isochema Ltd., is a highly specialized instrument for conducting gas sorption experiments. A precision-controlled water bath maintains stable temperatures within the reaction chamber, reaching 70 °C in this study. This is achieved through an integrated water jacket system, which evenly distributes heat to ensure consistent thermal conditions throughout the chamber. At the core of the reaction chamber, the ionic liquid (IL) is placed as the sorption medium, and ethane (C_2_H_6_) gas is introduced to assess its absorption behavior.

Pressure-regulation systems accurately control the gas-injection process, ensuring the desired experimental conditions are met. A Mass Flow Controller (MFC) regulates the ethane flow rate into the chamber, maintaining steady and uniform delivery for reliable measurements. The IGA-003 microbalance precisely measures minute changes in the IL’s mass during ethane sorption, yielding critical data on absorption trends. To account for external factors—such as the weight of the apparatus, buoyancy effects, and the supporting components—a counterbalance mechanism isolates and compensates for these variables, enabling precise mass measurements of the IL alone.

The system operates under an automated Data Acquisition (DAQ) and control platform, seamlessly managing key parameters such as gas flow, temperature, pressure, and mass changes. This level of automation ensures accuracy, repeatability, and efficiency, making the IGA-003 a robust and reliable tool for evaluating ethane sorption properties in ionic liquids.

### 4.4. Thermodynamic Simulation and Modelling

We employed the Peng-Robinson Equation of State (PR EoS), as shown in Equation (2), a widely recognized equation of state noted for its reliability in predicting the phase characteristics of fluid systems, particularly under high-pressure and temperature conditions. Using this Equation of state, we estimated the absorption of C_2_H_6_ across pressures up to 1.2 MPa and temperatures up to 70 °C. Selecting the PR EoS was intentional, as it effectively incorporates the critical properties of the gaseous and liquid components, ensuring alignment with the objectives and precision required for this study.

(2) $$P=\frac{RT}{v-b_{m}}-\frac{a_{m}(T)}{v\left(v+b_{m}\right)+b_{m}\left(v-b_{m}\right)}$$

The coefficients of this Equation of state were predicted using three (3) distinct mixing rules, as stated below [9]:

- van der Waals one single binary interaction parameter
- van der Waals two binary interaction parameters
- NRTL model combined with Wong-Sandler mixing rules (WS-NRTL)
- van der Waals mixing rules

Van der Waals proposed two (2) distinct mixing rules, vdW1 and vdW2, which were applied to predict the mixture parameters a_m_ and b_m_ [9]. The vdW1 mixing rule involves only one binary interaction parameter, l_ij_, whereas the vdW2 mixing rule requires two interaction parameters: l_ij_ and k_ij_.

The a_m_ parameter for both rules was calculated using Equation (3), with the temperature-dependent interaction parameter k_ij_ employed to determine a_ij_ as shown in Equation (6). The co-volume factor bm was derived through Equation (4) for vdW2 and Equation (5) for vdW1. Additionally, the interaction parameter b_F_ was estimated using Equation (7).

(3) $$a_{m}=\sum_{i}\sum_{j}x_{i}x_{j}a_{ij}$$

(4) $$b_{m}=\sum_{i}x_{i}b_{i}$$

(5) $$b_{m}=\sum_{i}\sum_{j}x_{i}x_{j}b_{ij}$$

(6) $$a_{ij}=\sqrt{a_{ii}a_{jj}}\left(1-k_{ij}\right)$$

(7) $$b_{ij}=\frac{b_{i}+b_{j}}{2}\left(1-l_{ij}\right).$$

#### Wong-Sandler Mixing Rule with NRTL Model (WS-NRTL)

This study employed the Wong-Sandler (WS) mixing rule to estimate the liquid and gas mixture’s attractive force parameter ‘a’ and co-volume parameter ‘b’, as shown in Equations (8) and (9). The Non-Random Two-Liquid (NRTL) mixing model was integrated to calculate the activity coefficients and Excess Gibbs free Energy using Equations (14)–(20).

Key binary interaction parameters (*τ*_ji_, *τ*_ij_, *τ*_ij__k_ij_) were utilized to determine mixture properties, where *τ*_ji_ and *τ*_ij_ denote the NRTL interaction parameters, and *g*_ij_ and *g*_jj represent the interaction Gibbs free energies between components ‘i’ and ‘j’ [9]. Following Nath and Henni [12], the non-randomness parameter, α, in Equations (15) and (16) was set to 0.3 for this research.

(8) $$a=RT\left(\frac{QD}{1-D}\right)$$

(9) $$=\left(\frac{Q}{1-D}\right)$$

(10) $$\;\;\;\;\;=\sum_{i}\sum_{j}x_{i}x_{j}b_{ij}\left(b-\frac{a}{RT}\right)$$

(11) $$D=\sum_{i}x_{i}\left(\frac{a_{i}}{b_{i}RT}\right)+\left(\frac{G^{ex}}{CRT}\right)$$

(12) $$C=-ln\;ln\;\left(\left(1+\sqrt{2}\right)/\sqrt{2}\right)$$

(13) $${\left(b-\frac{a}{RT}\right)}_{ij}=\frac{1}{2}\left[\left(b_{i}-\frac{a_{i}}{RT}\right)+\left(b_{j}-\frac{a_{j}}{RT}\right)\right]\left(1-k_{ij}\right)$$

(14) $$\frac{G^{ex}}{RT}=x_{i}x_{j}\left(\frac{\tau_{ji}G_{ji}}{x_{i}+x_{j}G_{ji}}+\frac{\tau_{ij}G_{ij}}{x_{j}+x_{i}G_{ij}}\right)$$

(15) $$G_{ij}=exp(-\alpha_{ij}\tau_{ij})$$

(16) $$G_{ji}=exp(-\alpha_{ij}\tau_{ji})$$

(17) $$\tau_{ij}=\frac{\left(g_{ij}-g_{jj}\right)}{RT}$$

(18) $$\tau_{ji}=\frac{\left(g_{ji}-g_{ii}\right)}{RT}$$

(19) $$ln\;\gamma_{i}=x_{j}^{2}\left[\tau_{ji}{\left(\frac{G_{ji}}{x_{i}+x_{j}G_{ji}}\right)}^{2}+\frac{\tau_{ij}G_{ij}}{{\left(x_{j}+x_{i}G_{ij}\right)}^{2}}\right]$$

(20) $$ln\;\gamma_{j}=x_{i}^{2}\left[\tau_{ij}{\left(\frac{G_{ij}}{x_{j}+x_{i}G_{ij}}\right)}^{2}+\frac{\tau_{ji}G_{ji}}{{\left(x_{i}+x_{j}G_{ji}\right)}^{2}}\right]$$

### 4.5. Critical Properties Calculations

Effective model utilization requires accurate thermo-critical properties of both the gas and the ionic liquid solvent. In this research, the Modified Lydersen-Joback-Reid (LJR) group contribution model [14] was applied to determine the thermocritical temperature (Tc), acentric factor ω, and critical pressure (Pc) for the ionic liquids (ILs). Table 7 summarizes these critical properties.

### 4.6. Optimizing Binary Interaction Parameters

The thermodynamic models and equations used in this study were initially developed by Nath and Sumon in Henni et al. [15] using MATLAB software, employing a bubble point algorithm. The optimization of binary interaction parameters, as defined in Equation (21), was performed using the Nelder–Mead simplex approach. Specifically, the ‘fminsearch’ function in MATLAB was applied to reduce the error in the objective Equation. The optimization was restricted to a pressure range of 0.1 to 1.2 MPa to mitigate the potential inaccuracies associated with experimental data at lower pressures.

(21) $$Err=\frac{100}{n}\sum_{i=1}^{n}\left[\frac{P_{Exp,i}-P_{Cal,i}}{P_{Exp,i}}\right]$$

### 4.7. Henry’s Law Constant, Enthalpy of Absorption, and Entropy of Solvation

The Henry’s Gas Law constant (H) for the three ILs studied was determined by evaluating the gradient/slope of the second-order polynomial obtained from plotting the mole fractions (x) of Ethane against the fugacity (f) of Ethane (C_2_H_6_) at the research temperatures. This gas constant, represented as H_i_, denotes the ratio of the solute’s fugacity (fi) to its mole fraction in the solvent (xi) at infinite dilution, evaluated at a specific temperature within a defined pressure range. In this study, $f_{i}^{V}\;and\;f_{i}^{L}$ denote the solute’s fugacity in the vapor and liquid phases, respectively, while $y_{i}$ and $x_{i}$ correspond to the mole fractions of Ethane in the liquid and vapour phases, as described by Huseynov [16].

Once Henry’s Gas Law constants (H) were evaluated from the solubility data, the entropy of solvation (ΔS^∞^) and the Enthalpy of absorption (ΔH^∞^) at infinite dilution for Ethane (C_2_H_6_) were evaluated using the corresponding thermodynamic equations.

(22) $$H_{i}=\underset{x_{i}\to 0}{\lim}\frac{f_{i}^{L}\left(T,P,x_{i}\right)}{x_{i}}=\underset{x_{i}\to 0}{\lim}\frac{f_{i}^{V}\left(T,P,y_{i}\right)}{x_{i}}$$

(23) $$∆H^{\infty }=R{\left(\frac{\partial \left(\ln H\right)}{\partial \left(1/T\right)}\right)}_{P}$$

(24) $$∆S^{\infty }=-R{\left(\frac{\partial \left(\ln H\right)}{\partial \left(\ln T\right)}\right)}_{P}$$

## 5. Conclusions

The experimental results provide valuable insights into the absorption behavior of Ethane in three structurally distinct ionic liquids. The key findings of this study can be summarized as follows:

- The alkyl chain length significantly affects ethane absorption by increasing its nonpolarity. IL-2, with its longer hexadecyl chain, demonstrated the highest solubility capacity, while IL-1, with its shorter alkyl chain, showed the lowest capacity for Ethane. This behavior was confirmed by a molecular simulation study [11].
- The type of cation also influences ethane solubility, but its effect is less pronounced than the alkyl chain length.
- Compared to conventional solvents, the studied ILs exhibit higher ethane absorption, which may limit their use in selective CO_2_-capture applications without further modifications.

In conclusion, while IL-2 showed the highest absorption capacity for Ethane, IL-1 appears more suitable for scenarios requiring lower ethane solubility. These findings emphasize the need for continued research into designing and optimizing ionic liquids to balance gas solubility and selectivity for industrial gas-separation processes. Future work could explore anion modifications or functionalized cations to improve CO_2_/ethane selectivity and reduce ethane co-absorption.

## Figures and Tables

**Table 1:** (**a**) C_2_H_6_ solubility data in IL-1; (**b**) C_2_H_6_ solubility data in IL-2; (**c**) C_2_H_6_ solubility data in IL-3.

(**a**) **IL-1**					
**303.15 K**		**323.15 K**		**343.15 K**	
**xC_2_H_6_**	**Pressure (MPa)**	**xC_2_H_6_**	**Pressure (MPa)**	**xC_2_H_6_**	**Pressure (MPa)**
0.021	0.0999	0.018	0.0999	0.014	0.0999
0.040	0.1998	0.034	0.1992	0.026	0.1920
0.059	0.3000	0.048	0.2987	0.038	0.2978
0.078	0.3990	0.061	0.3994	0.049	0.4001
0.095	0.4989	0.074	0.5002	0.060	0.4993
0.112	0.6021	0.087	0.5987	0.071	0.6001
0.129	0.7029	0.103	0.7306	0.082	0.7036
0.145	0.8000	0.110	0.7994	0.092	0.7990
0.161	0.8900	0.124	0.8995	0.103	0.9000
0.176	1.0034	0.139	0.9998	0.113	1.0004
0.188	1.0999	0.152	1.0993	0.123	1.1000
0.199	1.2000	0.166	1.1993	0.132	1.2000
(**b**) **IL-2**					
**323.15 K**		**333.15 K**		**343.15 K**	
**xC_2_H_6_**	**Pressure (MPa)**	**xC_2_H_6_**	**Pressure (MPa)**	**xC_2_H_6_**	**Pressure (MPa)**
0.024	0.0999	0.020	0.0999	0.018	0.0999
0.046	0.1998	0.037	0.1992	0.036	0.2001
0.067	0.3000	0.055	0.2987	0.054	0.3002
0.088	0.3990	0.070	0.3994	0.071	0.3999
0.107	0.4990	0.084	0.5002	0.088	0.4998
0.126	0.6021	0.099	0.5987	0.104	0.6026
0.145	0.7029	0.117	0.7306	0.119	0.7020
0.163	0.8000	0.125	0.7994	0.133	0.7999
0.181	0.8900	0.140	0.8995	0.146	0.8890
0.197	1.0034	0.156	0.9998	0.161	1.0014
0.210	1.1000	0.171	1.0993	0.174	1.0991
0.222	1.2000	0.186	1.1993	0.188	1.1997
(**c**) **IL-3**					
**303.15 K**		**323.15 K**		**343.15 K**	
**xC_2_H_6_**	**Pressure (MPa)**	**xC_2_H_6_**	**Pressure (MPa)**	**xC_2_H_6_**	**Pressure (MPa)**
0.026	0.0997	0.023	0.0999	0.022	0.0999
0.052	0.1999	0.044	0.1995	0.036	0.1989
0.077	0.3005	0.063	0.3010	0.051	0.3005
0.101	0.3989	0.081	0.4001	0.067	0.4007
0.123	0.4996	0.098	0.4996	0.080	0.4998
0.144	0.6012	0.115	0.5996	0.094	0.5997
0.164	0.6998	0.131	0.7009	0.107	0.7002
0.183	0.7999	0.148	0.8008	0.123	0.8006
0.206	0.9013	0.162	0.9007	0.136	0.8998
0.220	1.0001	0.176	1.0006	0.147	1.0002
0.237	1.0996	0.196	1.1235	0.160	1.0989
0.253	1.2020	0.208	1.2155	0.171	1.2036

Standard uncertainty u (x) = 0.006; Standard uncertainty u (T) = 0.1 K; Standard uncertainty u (P) = 0.0008 MPa.

**Table 2:** Estimated binary interaction parameters estimated for (**a**) vdW1, (**b**) vdW2, and (**c**) WS-NRTL along with their respective Average Absolute Deviation percentages (AAD %).

(**a**)							
**Ionic Liquids + C_2_H_6_**		**Temperature (°C)**	**Binary Interaction Parameter (k_12_)**				**% AAD**
IL-1		30	0.0251				3.16
		50	0.0175				4.65
		70	0.0076				3.82
IL-2		50	−0.0619				3.68
		60	−0.0486				5.15
		70	−0.0779				2.08
IL-3		30	−0.0180				3.60
		50	−0.0316				5.43
		70	−0.0452				6.25
(**b**)							
**Ionic Liquids + C_2_H_6_**	**Temperature (°C)**		**Binary Interaction Parameter**				**% AAD**
			**(k_12_)**		**(l_12_)**		
IL-1	30		0.0639		0.0081		1.05
	50		0.0962		0.0165		3.13
	70		0.1003		0.0178		1.63
IL-2	50		−0.0203		0.0090		1.05
	60		0.0260		0.0161		3.06
	70		−0.0412		0.0072		0.77
IL-3	30		0.0186		0.0066		0.91
	50		0.0423		0.0131		1.27
	70		0.0385		0.0143		2.58
(**c**)							
**Ionic Liquids + C_2_H_6_**		**Temperature (°C)**	**Binary Interaction Parameter**				**% AAD**
			**(k_12_)**	**(τ_12_)**		**(τ_21_)**	
IL-1		30	0.8971	2.9264		−1.1653	1.05
		50	1.1539	−0.5388		0.2736	3.19
		70	1.1676	−0.5310		0.2201	1.64
IL-2		50	0.8093	0.0347		−0.5349	1.05
		60	0.8842	−0.7371		0.3785	2.96
		70	0.8040	0.5633		−0.9996	0.73
IL-3		30	0.9710	0.6134		−0.8364	0.75
		50	0.9978	−0.4426		0.0021	1.28
		70	0.9771	−0.4671		0.0060	2.56

$k_{12}$ = $k_{21}$; $l_{12}$ = $l_{21}.$

**Table 3:** Entropy, Henry’s law constant (H), and Enthalpy of solvation between C_2_H_6_ and ILs.

Ionic Liquid (ILs)	Henry’s Law Constant (MPa)			$∆H^{\infty }$ (kJ/mol)	$∆S^{\infty }$ (kJ/Kmol·K)
IL-1	T = 30 °C	T = 50 °C	T = 70 °C		
	4.80	5.70	7.28	−8.97	−27.92
IL-2	T = 50 °C	T = 60 °C	T = 70 °C		
	4.13	4.98	5.21	−10.79	−32.33
IL-3	T = 30 °C	T = 50 °C	T = 70 °C		
	4.58	5.29	6.35	−7.04	−21.89

**Table 4:** CO_2_/C_2_H_6_ selectivity of the studied ILs.

Temperature (K)	IL-1	IL-2	IL-3
IL-1 & IL-3 at 303.15|IL-2 at 323.15	1.7	1.2	1.7
IL-1 & IL-3 at 323.15 K|IL-2 at 333.15	1.5	1.3	1.4
343.15	1.5	1.2	1.2
Average Selectivity	1.6	1.2	1.4

**Table 5:** Data on ionic liquids was used in this research.

Ionic Liquid	Chemical Formula	Acronym	Chemical Structure
1-Decyl-3-methylimidazolium bis (trifluoromethylsulfonyl imide) (≥98.0%)	C_15_H_26_F_6_N_3_O_4_S_2_	IL-1	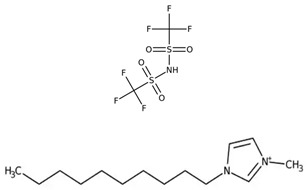
1-Hexadecyl-3-methyl imidazolium bis (trifluoromethylsulfonyl imide) (≥98.0%)	C_21_H_40_F_6_N_3_O_4_S_2_	IL-2	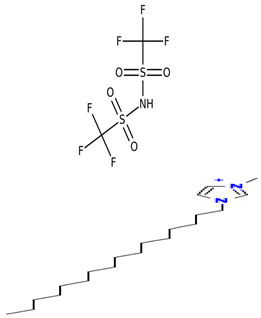
Triethyl tetradecyl ammonium bis (trifluoromethylsulfonyl imide) (≥98.0%)	C_20_H_44_F_6_N_2_O_4_S_2_	IL-3	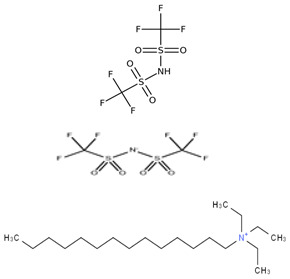

**Table 6:** Density values of the three ionic liquids used in this study.

Temperature (K)	Density (g/cm^3^)		
	IL-1	IL-2	IL-3
303.15	1.2727	-	1.1608
308.15	1.2684	-	1.1569
313.15	1.2640	-	1.1529
318.15	1.2597	-	1.1488
323.15	1.2553	1.1693	1.1446
328.15	1.2510	1.1652	1.1405
333.15	1.2465	1.1612	1.1362
338.15	1.2418	1.1572	1.1317
343.15	1.2368	1.1532	1.1270

Standard uncertainty u (ρ) = 0.0002 g/cm^3^; Standard uncertainty u (T) = 0.01 K.

**Table 7:** Critical properties of C_2_H_6_ and the ionic liquids used in this study.

Component	Tc (K)	Pc (Bar)	ω
IL-1	1345.1	18.700	0.5741
IL-2	1195.4	18.347	0.9176
IL-3	1207.7	14.265	1.1367
C_2_H_6_	305.4	48.8	0.099

## Data Availability

All data obtained in this study are reported in the manuscript.

## References

[1] Peng B., Zhao Y., Elahi E., Wan A. (2023). Can third-party market cooperation solve the dilemma of emissions reduction? A case study of energy investment project conflict analysis in the context of carbon neutrality. Energy.

[2] Dubey A., Arora A. (2022). Advancements in carbon capture technologies: A review. Clean. Prod..

[3] Freemantle M. (1998). Designer solvents-ionic liquids may boost clean technology. Chem. Eng. News Arch..

[4] Bahadur I., Phadagi R. (2019). Ionic Liquids as Environmental Benign Solvents for Cellulose Chemistry: A Review. Lond. IntechOpen.

[5] Vadillo J., Diaz-Sainz G., Gomez-Coma L., Garea A., Irabien A. (2022). Chemical and Physical Ionic Liquids in CO_2_ Capture Systems Using Membrane Vacuum Regeneration. Membranes.

[6] Sumon K.Z., Henni A. (2011). Ionic liquids for CO_2_ capture using COSMO-RS: Effect of structure, properties and molecular interactions on solubility and selectivity. Fluid Phase Equilibria.

[7] Quaye E., Henni A., Shirif E. (2024). Carbon Dioxide Solubility in Three Bis Tri (Fluoromethylsulfonyl) Imide-Based Ionic Liquids. Molecules.

[8] Florusse L., Peters S.R.C. (2008). High-pressure phase behavior of Ethane with 1-hexyl-3-methylimidazolium bis(trifluoromethylsulfonyl)imide. J. Chem. Eng. Data.

[9] Henni N., Husameldin I., Henni A. (2022). Solubility of Carbon Dioxide in Promising Ionic Liquids. Fluid Phase Equilibria.

[10] Rayer A., Henni A., Tontiwachwuthikul P. (2012). High-Pressure Solubility of Methane (CH_4_) and Ethane (C_2_H_6_) in Mixed Polyethylene Glycol Dimethyl Ethers (Genosorb1753) and Its Selectivity in Natural Gas Sweetening Operations. J. Chem. Eng. Data.

[11] Kurnia K.A., Matheswaran P., How C.J., Noh M.H., Kusumawati Y. (2020). A comprehensive study on the impact of chemical structures of ionic liquids on the solubility of Ethane. New J. Chem..

[12] Nath D., Henni A. (2020). Solubility of carbon dioxide (CO_2_) in four bis (trifluoromethylsulfonyl)imide ([Tf2N]) based ionic liquids. Fluid Phase Equilibria.

[13] Karunarathne S., Eimer D., Lars E. (2020). Density, Viscosity, and Excess Properties of MDEA + H_2_O, DMEA + H_2_O, and DEEA + H_2_O Mixture. Appl. Sci..

[14] Valderrama J., Rojas R. (2009). Critical properties of ionic liquids: Revisited. Ind. Eng. Chem. Res..

[15] Henni A., Tagiuri A., Nath D., Sumon S. (2017). Effect of Cation on the solubility of Ethane in three bis(fluorosulfonyl) Imide (FSI) Based Low viscosity Ionic Liquids. Fluid Phase Equilibria.

[16] Huseynov M. (2015). Thermodynamic and Experimental Studies of Ethane Solubility in Promising Ionic Liquids for CO_2_ Capture. Master’s Thesis.

